# Epidemiology of low-energy lower extremity fracture in Chinese populations aged 50 years and above

**DOI:** 10.1371/journal.pone.0209203

**Published:** 2019-01-14

**Authors:** Yanbin Zhu, Song Liu, Wei Chen, Bo Liu, Fei Zhang, Hongzhi Lv, Chenni Ji, Xiaolin Zhang, Yingze Zhang

**Affiliations:** 1 Department of Orthopaedic Surgery, The Third Hospital of Hebei Medical University, Shijiazhuang, Hebei, P.R. China; 2 Key Laboratory of Biomechanics of Hebei Province, Shijiazhuang, Hebei, P.R. China; 3 Department of Statistics and Epidemiology, Hebei Medical University, Shijiazhuang, Hebei, P.R. China; 4 Chinese Academy of Engineering, Beijing, P.R. China; International Medical University, MALAYSIA

## Abstract

This study aimed to investigate the epidemiology of low-energy lower-extremity fracture in Chinese men and women aged 50 years and above. This study was a part of Chinese National Fracture Survey (CNFS), which used the stratified multistage cluster random sampling method to recruit subjects between January and May 2015. A total of 512187 individuals participated in the CNFS and of them there were 154099 men and women aged 50 years and above included in this study for data analysis. Low-energy fracture was defined as a fracture caused by slip, trip or fall from standing height. Univariate analyses and gender-based multivariate logistic regression models were constructed to identify the independent risk factors. A total of 215 patients had sustained low-energy lower extremity fractures in 2014, indicating the overall incidence was 139.5 (120.9 to 158.2) per 100000 persons, with 127.8 (102.5 to 153.1) and 151.1 (123.8 to 178.5) per 100000 person-year in men and women. Over 80% of fractures occurred at home and on the common road. In men, alcohol consumption (OR, 2.00; 95%CI, 1.29 to 3.08), sleep duration<7h/d (OR, 2.60; 95%CI, 1.68 to 4.03) and history of past fracture (OR, 2.57; 95%CI, 1.33 to 4.95) were identified as significant risk factors associated with low-energy fractures. In women, advanced age (80+ years) (OR, 3.22; 95%CI, 1.80 to 5.75), alcohol consumption(OR, 1.72; 95%CI, 1.00 to 2.98), sleep duration <7h/d (OR, 2.11; 95%CI, 1.40 to 3.18), and history of past fracture (OR, 3.46; 95%CI, 1.97 to 6.09) were identified as significant risk factors and living in western region (OR, 0.60; 95%CI, 0.38 to 0.94) and current weight of 50 to 59.9 kg (OR, 0.17; 95%CI, 0.04 to 0.73) were identified as protective factors for fractures. Accordingly, awareness on the importance of sleep and alcohol consumption on fragility fracture should be improved, and health policies that focus on decreasing alcohol consumption and encouraging individuals to improve their sleep quality and duration should be considered. Maintaining a healthy bodyweight for women should be specifically emphasized to prevent low-energy fractures.

## Introduction

Osteoporosis-related fracture comprises a major part of public health care cost in the worldwide, and is associated high morbidity and mortality [1–3]. In 2001, the NIS Consensus Statement revised the definition of osteoporosis to include qualitative parameters related to low-energy fractures and set fracture prevention as the primary treatment goal for patients with osteoporosis [4]. In mid-aged and elderly individuals, approximately 70% of the osteoporosis-related fractures were lower extremity fractures, especially proximal femur and ankle fractures [5]. According to literature, the lifetime risk of lower extremity fractures caused by low-energy injury in Caucasian women was about 27%, and in men was about 10% [6, 7]. Moreover, elderly individuals who have osteoporosis-related lower or upper extremity fracture are at increased risk for secondary fall-related fractures and even death in the near future [8, 9].

By the end of 2016, there were over 280 million mid- and old-aged individuals aged 50 years and above in China (http://www.stats.gov.cn/tjsj/). Annually, approximately 1.4 million individuals had one low-energy lower extremity fracture in mid- and old-aged individuals [5]. It is predictable that this figure will increase dramatically in the next decade and accordingly, and the challenge from medical care costs is enormous for Chinese health policy-making institutions. Investigation of epidemiologic characteristics of these low-energy fractures is the fundamental task to develop public health programs. By use of these data on age- and gender specific incidence rates and the associated risk factors, we are able to adjust future policy, take preventive measures and optimize management in health care. Unfortunately, most epidemiologic reference data currently used by Chinese studies or policy-making institutions were from foreign researches. Data of such might not be applicable to our Chinese populations, due to the differences in ethnic origins, economic development and individual lifestyles between China and other countries. Additionally, most of previous studies focused on a single hospital, several hospitals in a region or populations of a certain subgroup [10–12], often reporting diverse incidence rates of low-energy fracture and controversial results for a certain risk factor.

To address these questions, we conducted this study with data available from the China National Fracture Survey (CNFS) database. In this study, we aimed to report the national population-based incidence rate of low-energy lower extremity fracture for overall populations and for different subgroups stratified by age, gender and et al; and to investigate the independent risk factors, in term of demographics, socioeconomics, geographical locations and individual lifestyles.

## Subjects and methods

CNFS was a population-based questionnaire survey, carried out in January to May of 2015, with aims to investigate the epidemiology of fractures in 2014. The survey was registered with the Chinese Clinical Trial Registry (ChiCTR-EPR-15005878). In our previous study [5], we described the details of the sampling methods (Supplementary **Fig 1**). Briefly, we used stratified multistage cluster random sampling method to recruit subjects. During the first phase, using stratified random sampling method we selected 8 provinces (municipalities) with 3 in eastern, 2 in central and 3 in western regions, based on geographic location, climate, population size and socioeconomic development. During the second phase, within each targeted province (municipalities), probability proportional to size method was used to complete the sampling separately in urban and rural areas. In each neighborhood or village, the households were calculated and selected. All members of eligible families living in their current residence at least 6 months were invited to participate in this study.

Our trained research team members were responsible for data collection, using the standardized questionnaires, and face-to-face interview with participants was conducted. Before data collection, informed consent was obtained from each participant. Data on demographics, geographical conditions, socioeconomics, and individual lifestyles were documented. Self-reported fractures between 1 January and December 2014 by participants and further confirmed by their clinical or radiographic data were confirmed. Eight quality control teams were responsible for check of random questionnaires (approximately 10% of all questionnaires) for potential omissions and errors. The CNFS was approved by the institutional review board of the third Hospital of Hebei Medical University.

### Current study

**Fig 1:**
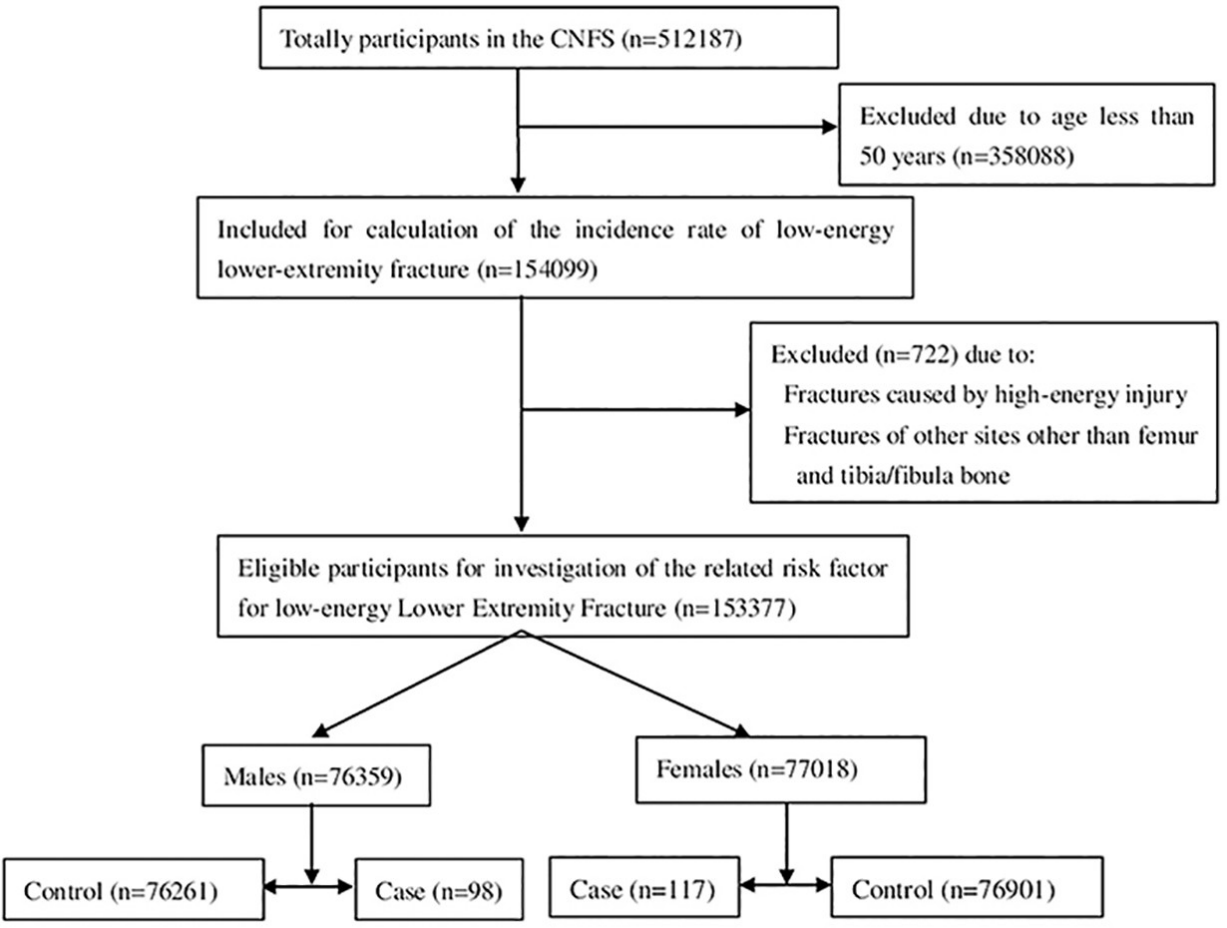
The flow chart for inclusion of the participants.

Low-energy fracture was defined as a fracture that was caused by slip, trip or fall from standing height. Fractures caused by high-energy injuries (traffic trauma, fall from height, crushing injury, sharp trauma and others) were excluded. A total of 154099 women and men aged 50 years and older participated in this study and 937 participants had at least one fracture of any site caused by either low-or high-energy in 2014. 215 patients reporting 217 cases of low-energy fractures of femur and tibia (fibula) were identified as case group. The remaining 153162 participants without any fracture were defined as control group. The detailed flow chart was presented in **Fig 1**.

Variables of interest included age, height, weight and body mass index (BMI), residency category (rural or urban), regions, latitude zone (20°-29.9°, 30°-39.9° or 40°-49.9°), ethnic origins, occupation, educational level, frequency of drinking intake (tea, carbonate beverages, coffee), smoking status, alcohol consumption, dwelling place (ground floor, > 2^nd^ floor with or without elevator), average sleep duration per day, history of past fracture, living situation (alone or with others), supplementation of calcium or Vitamin D or both for women and men; and age of menopause and number of births extra for women.

BMI was grouped on basis of the criteria suited to Chinese populations: underweight, <18.5 kg/m^2^; normal, 18.5–23.9 kg/m^2^; overweight, 24–27.9 kg/m^2^; obesity, > = 28 kg/m^2^ [5, 13]. Current smoking and alcohol consumption was defined as positive (yes), if participants smoked >1 time/week or drank > 1time/month during the 2014 year or the past year before fracture occurrence. Similarly within the time window, the average intake frequency of certain type of drinking was reported by participants; and supplementation of calcium or Vitamin D or both was defined as positive (yes) if participants acknowledged they take these medications for at least 1 month; otherwise, as negative (no).

### Statistical analysis

Incidence rates of low-energy lower extremity fractures were estimated by the number of fractures divided by the total number of overall population and population in each subgroup by age (5-year interval), as well as by demographic factors such as ethnic origin, geographical region, education, occupation, and residency category (rural or urban), stratified by gender. Differences in incidence rate between categories of nominal variables, such as ethnic origin, regions, occupation, and residency category, were tested using the Chi-square test. Age and education as ordered categorical variables and trends in incidence rates by them were tested by univariate logistic regression models. We also assessed incidence rates of low-energy femur or tibia (fibula) fractures based on sites (proximal, shaft and distal), in men or women or combined.

Two separate gender-based multiple logistic regression models were constructed to explore the independent risk factors associated with low-energy lower extremity fractures in women and men. Given the limited cases of fracture for each site (proximal, shaft and distal), we did not perform the subgroup analysis based on fracture site. The above-mentioned variables that were reported in previous literature to have positive or negative effects on bone mass density, or be associated with falls or low-energy fracture were all entered into the multivariate model. A stepwise backward elimination approach was used to exclude confounding covariates from the final model. Covariates were retained in the final model if the *p* value was ≤ 0.10. Odd ratio (OR) and 95% confidence interval (95% CI) were used to indicate the correlation magnitude between variables and the risk of low-energy fracture. The significance threshold was set at P < 0.05. The Hosmer–Lemeshow test was used to examine the goodness-of-ft of the final model, and a *p* value >0.05 indicated an acceptable fitness. All analyses were performed using SPSS 19.0 software (IBM Corporation, Armonk, NY)

## Results

**Fig 2:**
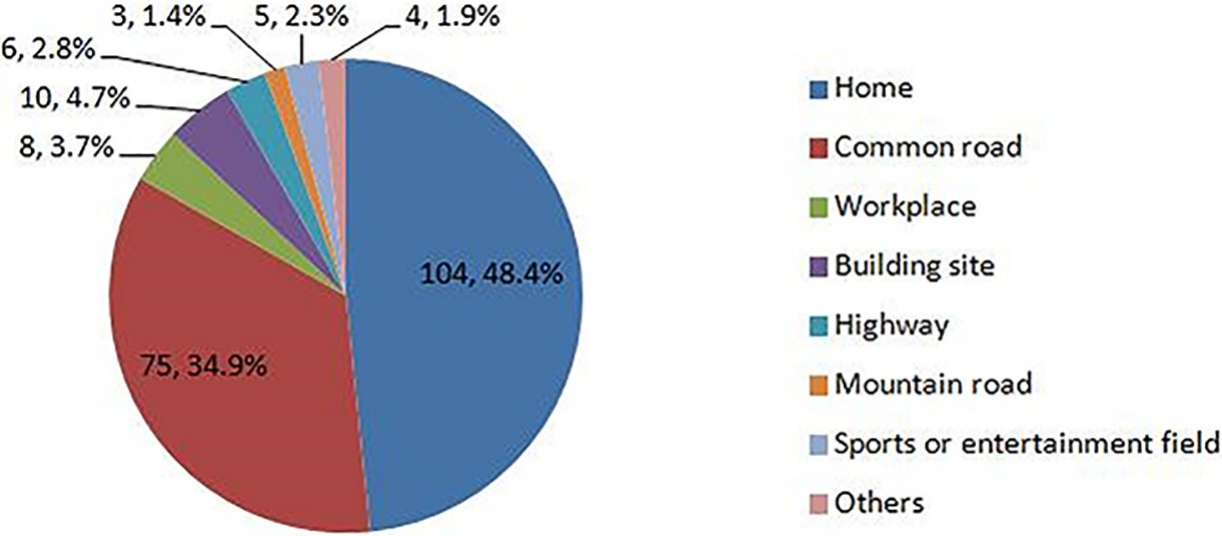
The detailed mechanism for low-energy fractures.

There were 76687 men and 77412 women, and their average age was 62.1 years (sd, 9.2). There were 70518, 30224 and 53357 participants from east, middle and west regions, respectively, and their average age was 62.5 (sd, 9.5), 62.2 (sd, 9.2) and 61.7 (sd, 9.0) years with significant difference (p<0.001). Of the 215 patients with 217 cases of fractures, there were 98 men and 117 women; and their average age at which fracture occurred was 65.1 years (sd, 10.2). Home (48.4%) was the most common place where lower extremity fracture occurred, followed by the common road (34.9%) (**Fig 2**). Totally, there were 87 patients with 88 cases of femur fracture and 128 patients with 129 cases of tibia (fibula) fracture.

The overall incidence rate of low-energy lower extremity fractures was 139.5 (120.9 to 158.2) per 100000 persons in 2014, with 127.8 (102.5 to 153.1) and 151.1 (123.8 to 178.5) per 100000 person-year in men and women, respectively (**Table 1**).

**Table 1:** National incidence of low-energy lower limb fractures in China by demographic, socioeconomic, and geographic factors in 2014.

	Sample size	Incidence (cases/100000 person-years) (95% CI)		
		Men	Women	Total
Individuals	154099	127.8 (102.5 to 153.1)	151.1 (123.8 to 178.5)	139.5 (120.9 to 158.2)
**Age**				
50–54	38849	91.5(49.3 to 133.8)	99(54.5 to 143.5)	95.2(64.6 to 125.9)
55–59	26114	103.1(47.1 to 159.1)	155.5(89.1 to 222)	130.2(86.5 to 173.9)
60–64	32854	104.4(54.8 to 154)	102.6(53.8 to 151.3)	103.5(68.7 to 138.3)
65–69	22032	193.2(110.6 to 275.7)	161.3(86.8 to 235.7)	177(121.5 to 232.5)
70–74	16713	142(61.7 to 222.3)	205.8(108 to 303.5)	173.5(110.4 to 236.6)
75–80	9275	186.8(64.9 to 308.7)	179.5(55.2 to 303.8)	183.3(96.2 to 270.3)
80+	8262	200.5(61.7 to 339.2)	398(209.2 to 586.9)	302.6(184.2 to 421)
p value for trend test		0.008	<0.001	<0.001
**Ethnicity**				
Han nationality	144433	128(101.9 to 154.2)	148.8(120.8 to 176.9)	138.5(119.3 to 157.7)
Other nationalities	9666	124.3(24.9 to 223.7)	186(64.6 to 307.3)	155.2(76.7 to 233.7)
p value for difference test		0.944	0.520	0.670
**Urbanization**				
Rural area	61294	104.3(68.2 to 140.5)	166.5(120.9 to 212.2)	135.4(106.3 to 164.5)
Urban area	92805	143.4(108.8 to 178)	141.1(107.1 to 175.1)	142.2(118 to 166.5)
p value for difference test		0.138	0.373	0.726
**Region**				
East	70518	135.5(97.2 to 173.8)	179.5(135.2 to 223.8)	157.4(128.1 to 186.7)
Central	30224	121(65.1 to 176.9)	162.9(99.1 to 226.7)	142.3(99.8 to 184.8)
West	53357	121.2(79.3 to 163.2)	107.6(68.4 to 146.7)	114.3(85.7 to 143)
p value for difference test		0.857	0.024	0.046
**Education**				
Illiterate	52109	154.1(104.5 to 203.8)	142.3(98.3 to 186.4)	147.8(114.8 to 180.7)
Primary school	54373	131.2(89 to 173.5)	156.6(108.7 to 204.5)	143.5(111.6 to 175.3)
Junior high school	42761	89(49 to 129)	168.2(113.3 to 223)	128.6(94.7 to 162.6)
Senior high school or above	4856	159.4(19.8 to 299)	0	103(12.8 to 193.2)
p value for trend test		0.141	0.920	0.322
**Occupation**				
Office worker	11203	117.9(36.3 to 199.6)	113.1(14 to 212.3)	116(53 to 179.1)
Farmer	57412	125.1(83 to 167.1)	132.3(91.4 to 173.3)	128.9(99.5 to 158.2)
Manual worker	29396	118.6(67.9 to 169.3)	85.6(32.6 to 138.6)	105.5(68.4 to 142.6)
Retired	30357	114.5(60.1 to 169)	219.1(145.6 to 292.7)	168(121.9 to 214.1)
Unemployed	17505	207.2(98.8 to 315.6)	195.4(111.9 to 278.9)	199.9(133.8 to 266.1)
Other	8226	117.5(2.4 to 232.5)	145.2(37.7 to 252.7)	133.7(54.8 to 212.7)
p value for difference test		0.585	0.059	0.083

The results of trend test showed a significant increasing trend of incidence rate of low-energy lower extremity fracture with age both in men (*p* = 0.008) and women (*p<*0.001) (**Table** 1). In men, individuals aged 80+ years had the highest incidence rate (200.5/100000 person-year), followed by those of 65–69 years (193.2/100000 person-year) and 75–79 years (186.8/100000 person-year). Similarly, women of 80+ years had the highest incidence rate (398/100000 person-year), followed by 75–79 years (179.5/100000 person-year) and 65–59 years (161.3/100000 person-year). Women living in eastern region of China had the highest incidence rate of low-energy lower extremity fractures; the difference of incidences among populations in 3 regions was significant statistically for women (p = 0.024), but non-significant for men (*p* = 0.857). Regarding other variables, there was not significant difference, such as Ethnicity, residency category, education, and occupation. The detailed information was presented in **Table 1**.

**Table 2** presented the incidence rates of femur and tibia (fibula) fractures, stratified by fracture site (proximal, shaft, distal). The overall incidence rate was 56.7 (44.6 to 68.8)/100000 person-years for femur fractures, and 83.0 (68.7 to 97.4)/10000 person-years for tibia (fibula) fractures. Proximal femur fractures accounted for approximately 80% of the total femur fractures, and their incidence was 45.0 (35.4 to 54.6) per 100000 person-years. Distal tibia and ankle fractures accounted for 74.3% of the total tibia (fibula) fractures, and their incidence rate was 61.7 (49.3 to 74.1) per 100000 person-years.

**Table 2:** National incidence of low-energy lower limb fractures in China by gender in 2014.

Item	Incidence rate per 1000000 population (95% CI)		
	Men	Women	Total
Femur	57.4 (40.4 to 74.3)	55.5 (38.9 to 72.1)	56.5 (44.6 to 68.3)
Proximal	49.6 (33.8 to 65.3)	42.6 (28.1 to 57.2)	46.1 (35.4 to 56.8)
Shaft	7.8 (1.6 to 14.1)	9 (2.3 to 15.7)	8.4 (3.9 to 13)
Distal	0	3.9 (0 to 7.8)	1.9 (0 to 3.8)
Tibia and fibula	70.4 (51.6 to 89.2)	95.6 (73.8 to 117.4)	83.1 (68.7 to 97.4)
Proximal	11.7 (4.1 to 19.4)	10.3 (3.2 to 17.5)	11 (5.8 to 16.3)
Shaft	6.5 (0.8 to 12.2)	14.2 (5.8 to 22.6)	10.4 (5.3 to 15.5)
Distal and ankle	52.2 (36 to 68.3)	71 (52.3 to 89.8)	61.6 (49.3 to 74)

### Multivariate analysis

After adjustment for confounding variables, alcohol consumption, sleep duration<7h/d and history of past fracture were identified as significant risk factors associated with low-energy lower extremity fractures in men (**Table 3**). The Hosmer–Lemeshow test demonstrated the adequate fitness (X^2^ = 7.105, *p* = 0.130).

**Table 3:** Results of multivariate logistic regression of risk factors for low-energy lower extremity fractures in men and women.

Variables	OR	95%CI		*P*
		Lower limit	Upper limit	
**Men**				
Alcohol consumption	2.00	1.29	3.08	0.002
Sleep duration <7h/d	2.60	1.68	4.03	<0.001
History of previous fracture	2.57	1.33	4.95	0.005
**Women**				
Age (years)				
50 to 64	Reference			
65 to 79	1.49	0.97	2.28	0.066
≥80	3.22	1.80	5.75	<0.001
Weight (kg)				
<40	Reference			
40 to 49.9	0.24	0.05	1.08	0.063
50 to 59.9	0.17	0.04	0.73	0.017
60 to 69.9	0.31	0.07	1.31	0.111
70 to 79.9	0.23	0.05	1.17	0.083
≥80	0.40	0.07	2.29	0.303
Region				
East	Reference			
Middle	0.88	0.55	1.40	0.578
West	0.60	0.38	0.94	0.026
Alcohol consumption	1.72	1.00	2.98	0.050
Sleep time<7h/d	2.11	1.40	3.18	<0.001
Supplementation of calcium or VD or both	0.54	0.27	1.06	0.074
History of previous fracture	3.46	1.97	6.09	<0.001

In women, advanced age (80+ years), alcohol consumption, sleep duration <7h/d, and history of past fracture were identified to be associated with increased risk of lower extremity fractures. Living in west region (compared to eastern region) and weight of 50–59.9kg (compared to <40kg) were identified as protective factors for low-energy fracture. Supplementation of calcium or Vitamin D or both seemed to have positive effect on prevention of low-energy lower extremity fractures, but the significance did not approach to the statistical level (*p* = 0.074). The detailed statistical results were presented in **Table 3**. The Hosmer–Lemeshow test demonstrated the adequate fitness (X^2^ = 6.087, p = 0.638).

## Discussion

To our knowledge, this is the most representative and comprehensive population-based study carried out in China, in which the incidence rates of low-energy lower extremity fractures by demographic and socioeconomic factors were estimated and various risk factors were identified. Over 80% of the fractures occurred at home and on the common road, which emphasized the importance of the primary preventive measures, especially home prevention. We also observed a significant trend towards increasing incidence of lower extremity fracture with age in women, but not in men. After adjustment for confounding variables, alcohol consumption, sleep duration<7h/d and history of past fracture were identified as significant risk factors for low-energy fractures, both in women and men. Additionally in women, weight of 50 to 59.9 kg and living in west region were identified as protective factors for low-energy fractures.

### Comparison of incidences of low-energy fracture with studies

Knowledge of population-based incidence of low-energy fractures is fundamental to develop public health programs, but national epidemiologic surveys of such were very scarce. Currently, most researches selected a single hospital, a certain region or a subgroup of population as study object to obtain knowledge of epidemiologic situation [14–17]. In addition, geographical location, socioeconomic development, and individual lifestyles differ from each other among countries and or regions in the same country [18, 19]. Therefore, it might be explainable that the incidence rates of fractures in literature were greatly varied. In UK, Kaye et al [20] used the data from General Practice Research Database to report the incidence was 472 per 100000 person years for total lower extremity fracture, and 330/100000 person-years for femur and tibia (fibula) fractures, in individuals aged 50 years. In Finland, incidence of hip fracture was reported as 170 to 210 and 364 to 450 per 100000 person-years in men and women aged 50+ years [21, 22] and the incidence of low-energy ankle fracture was 114 and 174 per 100000 person-years for elderly men and women [23]. In USA, the adjusted annual incidence of hip fracture was 129 and 224 per 100000 person-years for men and women above 45 years [24]. Author also observed the declined trend of hip fracture incidence during the past 10 years before 2006, and suggested this result partially benefit from the active bone health intervention [24]. Compared to those western developed countries, the figures reported in this study was substantially lower, as 1/5to 1/3 of them. Even in Asian, the incidence of lower extremity fracture reported in this study was much lower than that of neighboring countries, as Japna [25] and South Korea [26]. The underlying mechanism might be related to relatively low prevalence of osteoporosis [27, 28]. Despite, low-energy lower extremity fracture caused undoubtedly constitutes a major public health issue in modern China, due to the huge base of mid-aged and elderly individuals (http://www.stats.gov.cn/tjsj/).

### Risk factors associated with the low-energy fractures

Alcohol consumption, insufficient daily sleep duration (<7h/d) and history of past fracture were identified as significant risk factors for low-energy lower extremity fracture, both in men and women. Clark et al [29] suggested excess consumption of alcohol increase the risk of fractures through metabolic effects and alcohol-related falls. As was previously reported [30], men consuming >8 units and women consuming >6 units of alcohol on the heaviest drinking day in the past week had the 1.65 to 2.07-time increased risk of lifetime fractures. Stone et al [31] and Holmberg [32] reported the adverse effects of insufficient sleep on the risk of fracture in men or women and suggested frequent fall caused by insufficient sleep was the direct reason for fracture occurrence. History of past fracture as a risk factor for low-energy fracture was well-established in literature, and accelerated bone loss and the increased tendency to fall after the first fracture were suggested to contribute to the secondary fracture in a near future [33, 34]. Therefore, awareness on the importance of sleep and alcohol consumption on fragility fracture should be improved, and health policies that focus on decreasing alcohol consumption and encouraging individuals to improve their sleep quality and duration should be considered to reduce and prevent the low-energy fractures.

In women, another modifiable factor was the weight, and in this study weight of 50 to 59.9kg was shown to have significantly positive effect on the lower extremity fracture, compared to the weight of <40kg. And this result was consistent with that of a previous meta-analysis [35], which showed being underweight increased the risk of lower extremity fractures, while being overweight increased the risk of upper arm fractures, in elderly women. Therefore, maintaining a healthy bodyweight with a normal BMI is of clear significance on prevention of low-energy fractures, for both upper and lower extremity fractures. Other several non-modifiable risk factors have also been identified to be associated with lower extremity fracture in women, including advanced age (80+ years) and geographic position. And the latter this might be related to intense pace of life and more frequent exposure to the hazardous lifestyles for individuals living in east region. However, this result this result should be treated cautiously and dialectically. On the hand, individuals in west region had a relatively shortened life span (72.3 years), (http://bbs1.people.com.cn/post/2/0/0/166947095.html), and it was a possibility that death came prior to the incident fracture, especially hip fracture. On the other hand, western region like Xinjiang province had a highest amount of solar radiation annually, which objectively facilitated the synthesis of vitamin D and calcium absorption to benefit bone health, especially in elderly individuals [36–38]. In addition, inhibition of alcohol due to religious reason among Muslim population in western part of China (Xinjiang area) and economy of that region being still centered on agriculture (higher physical activities) might be also the contributing factors [39].

### Strengths and limitations

The main strengths of the current study included the stratified multistage cluster random sampling method used for recruiting subjects, largest sample size, face-to-face interview for data collection and adjustment for numerous important covariates. “Double” identification of fracture case via patients' self-reports and clinical or radiographic data increased the accuracy and precision of diagnosis.

Despite, there are some potential limitations that should be mentioned. Firstly, there was some recall bias due to its nature of retrospective design. Secondly, there remain residual confounding effects, because data on clinical chronic disease status, medication use and other socioeconomic factors that may have affected BMD or propensity to fall could not be available for adjustment in the multivariate analysis model. Thirdly, the study could not capture information about fracture cases in which the patients died. Overall, the incidence rate of low-energy lower extremity fracture was underestimated.

## Conclusions

This study provided the distribution of low-energy lower extremity fractures, including population-based incidence, occurrence place, and the associated risk factors. These data could be used as updated clinical evidence base for healthcare planning and preventive efforts in China, as elsewhere. On the basis of these findings, awareness on the importance of sleep and alcohol consumption on fragility fracture should be improved, and health policies that focus on decreasing alcohol consumption and encouraging individuals to improve their sleep quality and duration should be considered. Specifically for women, maintaining a healthy bodyweight should be emphasized to prevent the lower extremity fractures.

## Supporting information

S1 Table The detailed data for males. (XLSX)

S2 Table The detailed data for females. (XLSX)

S1 Fig (TIF)
