# Supplementary figures and images for: Epidemiology of low-energy lower extremity fracture in Chinese populations aged 50 years and above
Source: PLoS One. 2019 Jan 14;14(1):e0209203. doi: 10.1371/journal.pone.0209203 (PMC6331176; doi:10.1371/journal.pone.0209203)

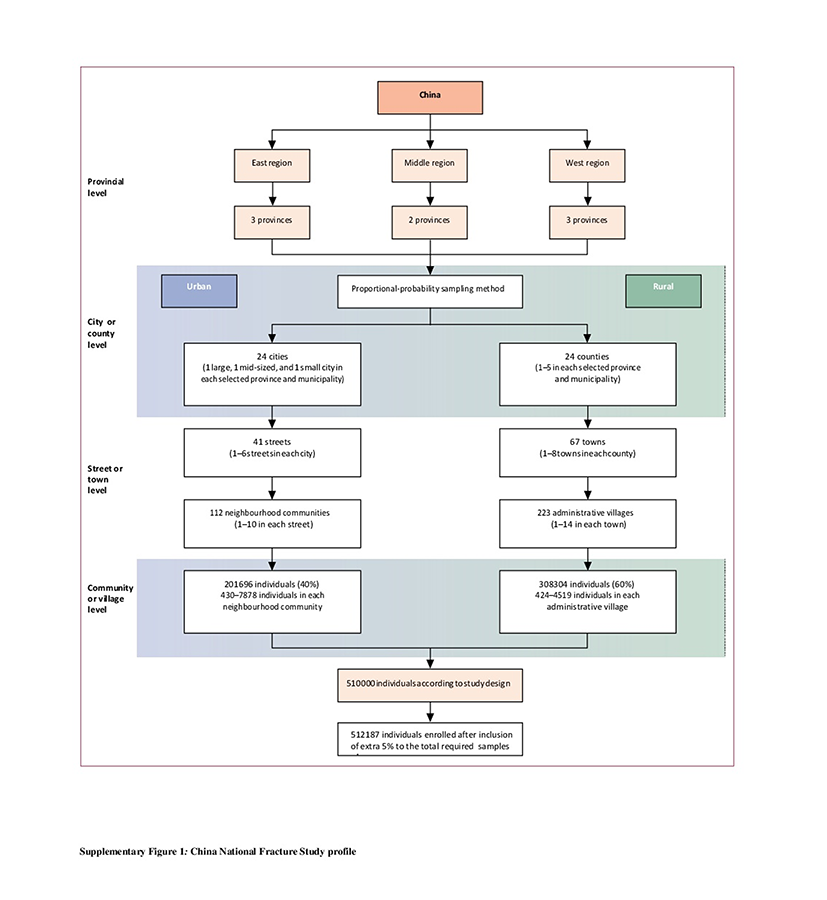

Supplement: S1 Fig — (TIF) [file pone.0209203.s003.tif]
